# Targeted Dendrimer-Coated Magnetic Nanoparticles for Selective Delivery of Therapeutics in Living Cells

**DOI:** 10.3390/molecules25092252

**Published:** 2020-05-10

**Authors:** Paola Parlanti, Adriano Boni, Giovanni Signore, Melissa Santi

**Affiliations:** 1 Center for Nanotechnology Innovation@NEST, Istituto Italiano di Tecnologia, Piazza San Silvestro 12, 56127 Pisa, Italy; paola.parlanti@rutgers.edu (P.P.); adriano.boni@iit.it (A.B.); 2 NEST, Scuola Normale Superiore, Piazza San Silvestro 12, 56127 Pisa, Italy; 3 Child Health Institute of New Jersey and Department of Neuroscience and Cell Biology, Rutgers Robert Wood Johnson Medical School, 89 French Street, New Brunswick, NJ 08901, USA; 4 Fondazione Pisana per la Scienza, via Ferruccio Giovannini 13, 56017 San Giuliano Terme (PI), Italy; 5 NEST Istituto Nanoscienze-CNR, Piazza San Silvestro 12, 56127 Pisa, Italy

**Keywords:** dendrimers, peptide aptamers, targeted drug delivery, magnetic nanoparticles

## Abstract

Nanoparticles are widely used as theranostic agents for the treatment of various pathologies, including cancer. Among all, dendrimers-based nanoparticles represent a valid approach for drugs delivery, thanks to their controllable size and surface properties. Indeed, dendrimers can be easily loaded with different payloads and functionalized with targeting agents. Moreover, they can be used in combination with other materials such as metal nanoparticles for combinatorial therapies. Here, we present the formulation of an innovative nanostructured hybrid system composed by a metallic core and a dendrimers-based coating that is able to deliver doxorubicin specifically to cancer cells through a targeting agent. Its dual nature allows us to transport nanoparticles to our site of interest through the magnetic field and specifically increase internalization by exploiting the T7 targeting peptide. Our system can release the drug in a controlled pH-dependent way, causing more than 50% of cell death in a pancreatic cancer cell line. Finally, we show how the system was internalized inside cancer cells, highlighting a peculiar disassembly of the nanostructure at the cell surface. Indeed, only the dendrimeric portion is internalized, while the metal core remains outside. Thanks to these features, our nanosystem can be exploited for a multistage magnetic vector.

## 1. Introduction

Nanomedicine aims at developing smart devices that are able to interact selectively with pathologic cells or tissues, allowing a better signal-to-noise ratio in diagnostics and improved therapeutic index in drug delivery applications. Many approaches have been proposed to achieve this goal, including the use of organic-based systems, polymeric nanoparticles, and dendrimers [1]. Among those, dendrimers are receiving increasing attention due to their controllable size and surface properties, which make them an attractive platform for drug delivery [2,3]. For example, it is known that polyamidoamine (PAMAM) dendrimers can adsorb small molecules noncovalently, releasing them in mildly acidic conditions, and this feature has already been exploited in the controlled delivery of fluorescent and therapeutic payloads [4]. Furthermore, dendrimer-based structures can be easily derivatized with targeting sequences such as peptides and oligonucleotides [5]. Dendrimers represent an excellent coating for metal nanoparticles, allowing the preparation of nanodevices that merge advantages of both organic and inorganic nanomaterials, such as high drug-loading capability and effective use as a contrast agent or as vector for magnetically targeted delivery. Not surprisingly, several hybrid structures composed of a metallic core and a dendrimer shell have been recently described [6,7,8], and some of them have intriguing capabilities as delivery agents. We recently reported the synthesis and intracellular behavior of self-assembled systems composed of a core magnetic nanoparticle and an amphiphilic dendrimer coating [7]. These structures have the unique advantage of combining features of superparamagnetic iron oxide nanoparticles (SPION) with the chemical flexibility of dendrimers, which can accommodate molecular payloads by noncovalent encapsulation and are easily functionalized with targeting sequences. One of the most interesting targets is the transferrin receptor (TfR) [9]. It is widely exploited for the delivery of therapeutics inside cells, especially in solid tumors and across the blood–brain barrier (BBB) [10,11]. Indeed, it was reported in the literature that TfR is overexpressed in most tumors that need iron to maintain high growth rates [12]. Furthermore, it is also one of the few receptors present on the BBB, and therefore, it can be used for the transport of payloads to the nervous system in the treatment of several pathologies [13]. Here, we describe our findings in the development and in vitro evaluation of targeted hybrid metal/organic structures for targeted pH-dependent delivery and highlight the peculiar “disassembly at cell surface” behavior of our system.

## 2. Results and Discussion

### 2.1. Synthesis and Characterization of Dend-NP-Coum

**Scheme 1:**
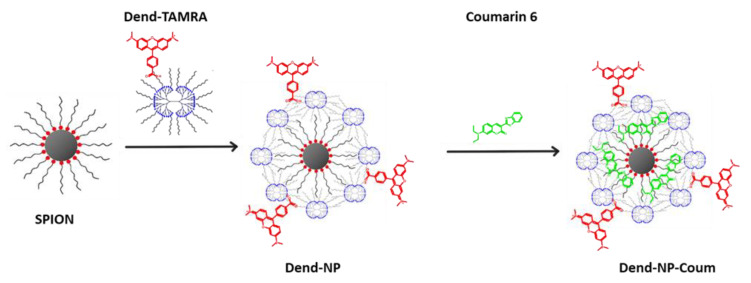
Synthetic strategy for the assembly of Dend-NP and Dend-NP-Coum.

First, we assembled the metal–organic scaffold (Dend-NP) using our already published protocols (Scheme 1) [7]. Briefly, we synthesized oleic-coated iron nanoparticles by thermal decomposition as described elsewhere [14]. Obtained nanoparticles had an average diameter of 8.3 nm. Then, iron nanoparticles were coated with PAMAM dendrimer (in particular, we used PAMAM dendrimers modified with lipid 2-hydroxydodecyl (C_12_) moieties with 48 amino hydrophilic functional groups and 16 hydrophobic C_12_ chains). Nanoparticles were characterized as described previously by us [7]. In this phase, we used a fluorescently labeled dendrimer, to allow for a better localization of the nanostructure in cells. Commercial amphiphilic PAMAM dendrimer (25% oleic acid coated) was derivatized at low extent (1.35 molecules/dendrimer) with the fluorescent reporter carboxytetramethylrhodamine (TAMRA). The reaction proceeded smoothly under classic conditions for *N*-hydroxysuccinimide (NHS)-based chemistry, and the product was easily purified from unreacted fluorophore by dialysis. Next, a dendrimer–SPION complex was formed in phase transfer conditions, leading to fluorescent Dend-NP. Then, we loaded the nanostructure with coumarin 6, which is a lipophilic dye chosen as model fluorescent molecules for small drugs. Encapsulation in the inner cavities of PAMAM dendrimer was eased by the hydrophobicity of coumarin 6. The unloaded dye was easily removed by magnetic washings followed by size exclusion chromatography on Sephadex resin. This strategy allows the efficient loading of unmodified drugs preserving the solubility and surface properties of the dendrimeric scaffold; additionally, this approach outperforms those based on drug conjugation due to the faster release of the drug once appropriate conditions, such as pH changes, are met. Hydrodynamic radius was measured by dynamic light scattering (DLS) and nanoparticles have an average diameter of 133 ± 10 nm. Loading extent of the obtained Dend-NP-Coum was determined by the spectrophotometric quantitation of coumarin concentration and inductively coupled plasma mass spectrometry (ICP-MS) determination of iron present in the sample. From these data, we obtained a coumarin/iron molar ratio of 2.5:1. Despite the high coumarin loading on the nanoparticles, fluorescence arising from the coumarin is unsurprisingly quite dim. Indeed, both the close proximity to the nanoparticle and the high local concentration of coumarin could sensibly decrease fluorescence emission. Additionally, the known strong solvatochromism typical of coumarin dyes [15,16] can further lower fluorescence emission due to the formation of polar hydrogen bonds within dendrimer cavities. Positively charged complexes were stable for up to 6 months, while a negative targeted nanostructure was stable for several weeks at 4 °C. After these times, nanoparticles started to aggregate and precipitate (the diameter of Dend-NP measured after 6 months was around 489 ± 63 nm).

### 2.2. Nanoparticles Internalization in Living Cells

**Figure 1:**
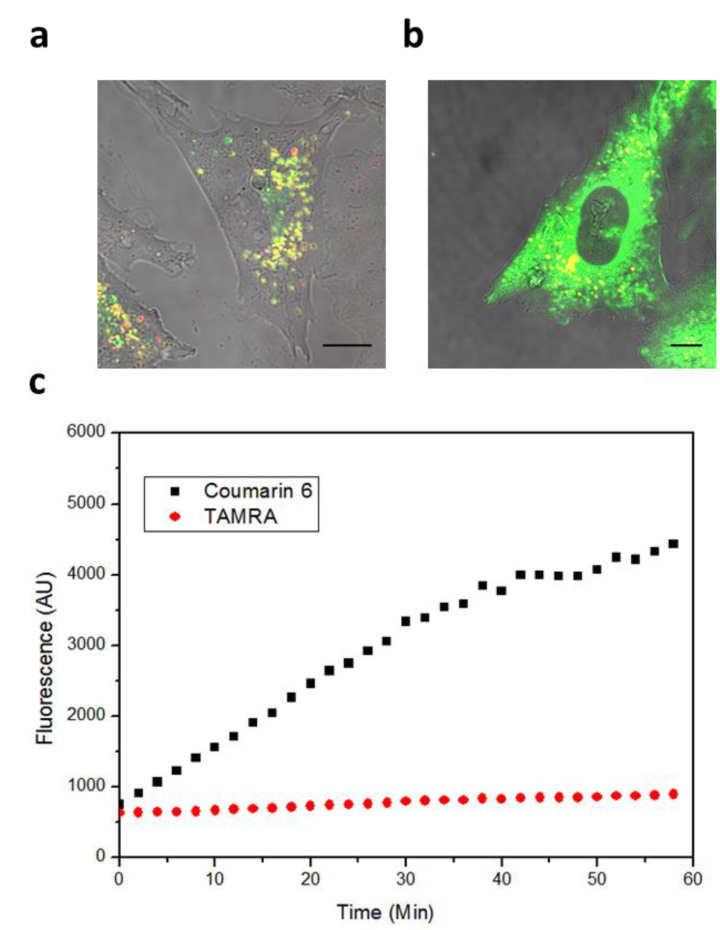
Nanoparticles internalization inside cells. (**a**) Intracellular distribution of Dend-NP shortly after (10 min) administration to the cells; (**b**) intracellular distribution of Dend-NP after 2 h; (**c**) Fluorescence increase of coumarin 6 (black) and carboxytetramethylrhodamine (TAMRA) (red) during the endocytosis process. Scale bar: 10 µm.

We administered Dend-NP-Coum to adenocarcinoma pancreatic cells (MIA PaCa-2), and we observed that the assembly readily internalizes owing to the presence of multiple positively charged amino groups on the surface of the dendrimers, leading to a typical vesicular signal, in agreement with our previous observations on the internalization of SPION–dendrimer assemblies (Figure 1a,b) [7,8]. The vesicular signal observed shortly after administration evidences poor release of the payload (Figure 1a), keeping with the essentially neutral pH of early endosomes. However, as endocytosis process evolves, the increasingly acidic milieu of the endocytic vesicles promotes protonation of the inner tertiary amines of dendrimer coating, leading to desorption of the membrane-permeable dye that diffuses into the cytoplasm (Figure 1b). This is evidenced by the increased coumarin fluorescence during the endocytosis process. The intracellular distribution of coumarin is typical of similar dyes [15], which mainly localize—and enhance their brightness—in lipophilic environments such as endoplasmic reticulum and intracellular vesicles. Interestingly, plotting the fluorescence signal arising from the coumarin against time indicates that the release of fluorescent payload from the nanostructure occurs with kinetics that are typical of the endocytosis process (Figure 1c). Fluorescence plateau, which is achieved after approximately 60 min from the administration, is in perfect agreement with a release occurring at endosomal level, [17] where pH can vary in the range of 4.8–6.8, depending on the stage of endocytosis process. The achievement of a nearly stationary phase might indicate equilibrium between coumarin release from the dendrimer scaffold and leakage from the cell in the surrounding medium [18]. Conversely, the signal from the labeled dendrimer remains strongly vesicular, indicating low or negligible endosomal escape of the nanostructure.

### 2.3. Nanoparticles Internalization under Magnetic Field

**Figure 2:**
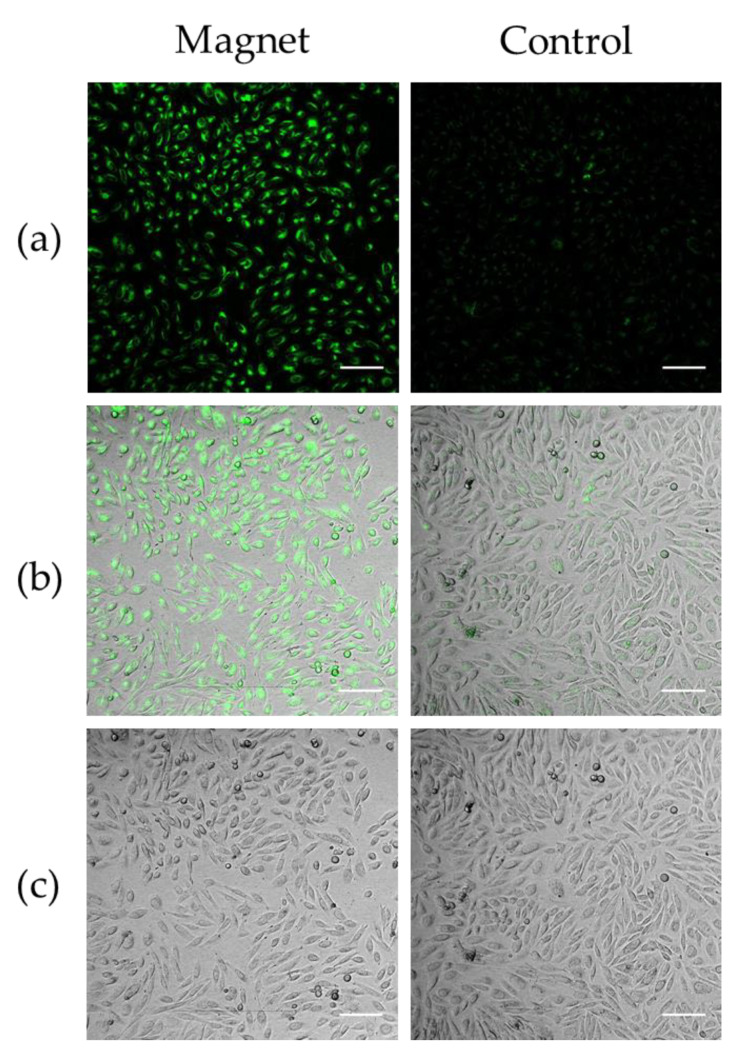
Internalization and coumarin release from Dendr-NP-Coum in the same dish. (**a**): fluorescence emission from coumarin 6. (**b**): overlay. (**c**): brightfield. Scale bar: 100 µm.

Then, we evaluated the capability of our nanostructure to preferentially internalize in cells under the guide of an external magnetic field. To this end, we first incubated Dend-NP-Coum in MIA-PaCa-2 cells, and then, the dish was placed on the microscope stage. Here, half of the dish (magnet) was placed over a 2 × 5 × 12 mm neodymium magnet, and nanoparticle incubation was performed in this condition for 30 min. The other half of the dish was used as a control in which nanoparticles incubation was carried out without any magnetic field. The magnet was removed after the incubation, and cells were washed and imaged alternatively on “magnet” and “control” fields using the same parameters (Figure 2). The increased concentration of nanoparticles caused by the magnetic field led to improved nanoparticle internalization and hence to an increase of released coumarin in the cytoplasm (average fluorescence intensity: 7200 ± 2100 counts on magnet side, 3500 ± 1200 on the control side, *n* = 14, *p* < 0.0002). An aspect that should be considered is that the magnet alone can force the internalization of nanoparticles in cells. This effect can be exploited to increase the local internalization of the nanoparticles only in the area stimulated by the magnetic field, decreasing either the side effects or the doses that have to be administered to patients.

### 2.4. Functionalization and Loading of Nanoparticles with Doxorubicin

**Scheme 2:**
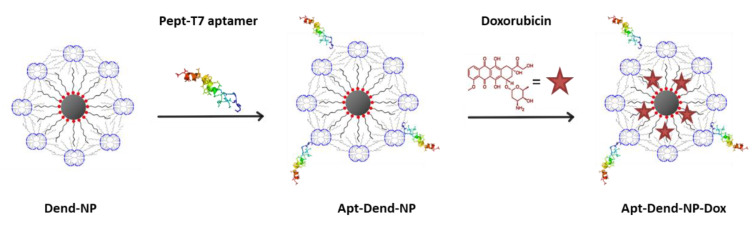
Synthetic strategy for the assembly of Apt-Dend-NP and Apt-Dend-NP-Dox.

**Figure 3:**
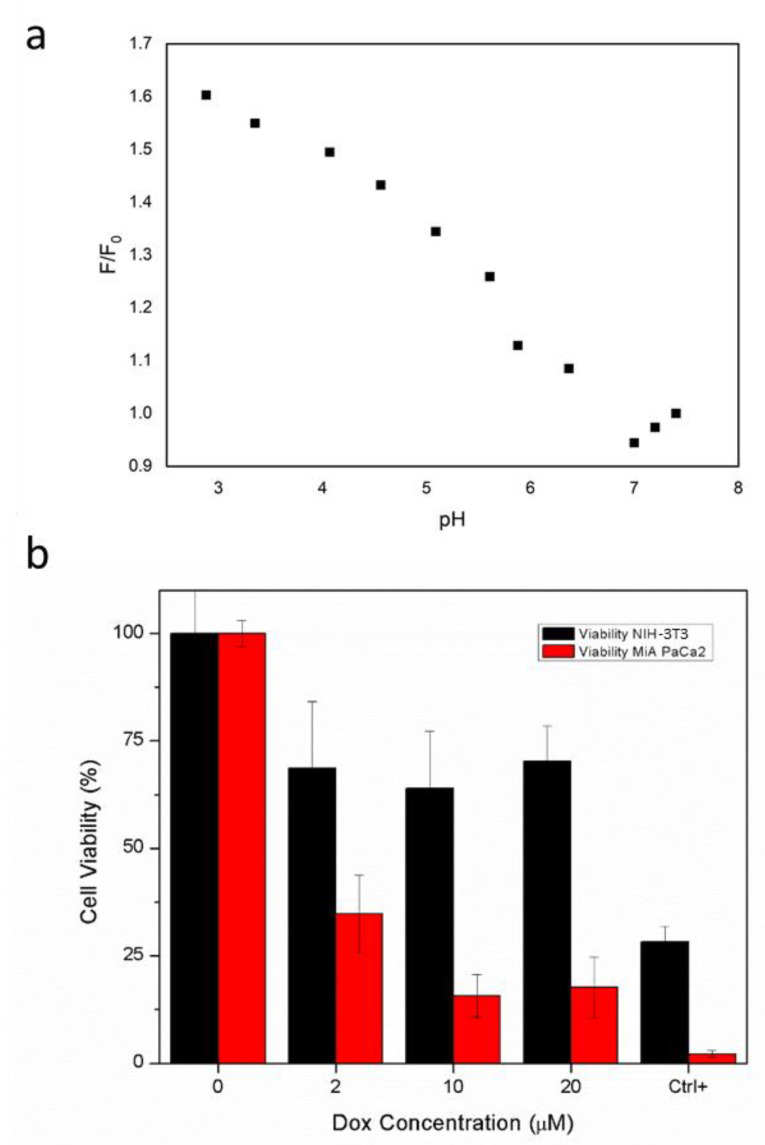
(**a**) Release of doxorubicin (Dox) from Apt-Dendr-NP in medium at different pH; (**b**) Cell viability after treatment with Apt-Dendr-NP.

Next, we examined the surface functionalization of Dend-NP and its capability to perform the targeted delivery of a therapeutic payload in cells. Realization of a targetable nanodevice for theranostics can significantly benefit from the inhibition of nonspecific uptake pathways, which could lead to off-target effects upon administration. To this end, we focused our attention on the presence of amino groups on the surface of the dendrimers. It is known that positively charged residues can improve adhesion to the plasma membrane, which is a critical step in any endocytosis process [19]. A suitable strategy to circumvent this issue could involve the derivatization of the positively charged amino residues with negative functional groups. In our vision, the most suitable functional group for dendrimer derivatization was represented by succinic anhydride. Derivatization with succinic anhydride has two main advantages over more commonly used polyethylene glycol (PEG) chains: (1) limited or absent masking effect of the targeting unit due to the small steric hindrance and (2) the absence of PEG chains should limit formation of anti-PEG immunoglobulin G (IgG), which is a common issue that is found during in vivo administration of PEGylated nanodevices [20]. Note that, additionally, the presence of tertiary amino groups in the dendrimer scaffold could lead to an effective zwitterionic system. Such systems have been reported as efficient antifouling agents that limit protein adsorption in blood [21]. Our targeting peptide of choice (Pept-T7, HAIYPRH) is reported as an effective and selective binding unit toward human TfR [22,23]. We focused on TfR owing to its reported overexpression in many solid tumors (pancreatic, breast, head/neck) and on the endothelium of the blood–brain barrier (BBB). We firstly conjugated this targeting sequence to the amino groups of the coating dendrimers by standard carbodiimide coupling performed in organic conditions with dimethyl sulfoxide (DMSO) [24]. The functionalization level was kept at low values (10%–20%) to avoid overderivatization and destabilization of the delicate hydrophobic/hydrophilic balance, which determines the stability of the metal nanoparticles. Next, we masked the remaining amino groups on the surface of the dendrimers by reaction with succinic anhydride. The resulting system was stable in aqueous solution. Finally, we assembled hybrid SPION–dendrimer nanoparticles according to our established protocol [7]. The resulting nanoparticles (Apt-Dend-NP, Scheme 2) were characterized by DLS and we shown to be rather monodisperse (hydrodynamic diameter: 132 ± 17 nm) and with negative surface charge (−18 ± 1 mV). Then, we sought to realize our drug-loaded nanoparticle assembly. To this end, we incubated our system with doxorubicin (Dox) to allow loading of the drug in the inner cavities of Apt-Dend-NP by hydrophobic interaction (Scheme 2). Not surprisingly, the pH-dependent release from the dendrimer cavities was fully retained in this new architecture: while thermal desorption at 37 °C at or above pH 7 did not show any detectable release of doxorubicin, leakage increased sensibly at lower pH (Figure 3a). In a cuvette analysis of doxorubicin fluorescence enhancement following acidification, which is a parameter that is correlated to dequenching and desorption from the dendrimer, [25] showed extensive release at acidic pH, with most of the drug desorbed at pH usually found in early-late endosomes or lysosomes (4.5–6). This behavior is in keeping with the reported pKa of inner tertiary amines of PAMAM dendrimers and demonstrates the suitability of Apt-Dend-NP for controlled release at the endolysosomal level. Notably, it is also in perfect agreement with the reported behavior of other PAMAM dendrimers, [26,27] showing that adsorption on the nanoparticle and the use of an amphiphilic PAMAM dendrimer does not affect encapsulation or release efficiency.

### 2.5. Cytotoxicity Assay in Living Cells

Finally, we evaluated the internalization and cytotoxic activity of the system in target and control cells. In this case, we used two different cell lines: a control one with a low expression of TfR (fibroblasts from mice embryo, NIH-3T3) and a tumoral one (MIA-PaCa-2) that instead presented overexpressed TfR on the cell membrane (Figure 3b). First, we observed that Dend-NP, which is only derivatized with negatively charged succinate residues, shows only minimal nonspecific uptake in cells (Figure S1). This result was expected, given the reportedly poor internalization of negative-carboxyl-terminated nanoparticles due to unfavorable electrostatic interaction with the cell membrane [21]. Conversely, the presence of targeting peptide T7 on derivatized Apt-Dend-NP triggers endocytosis, exploiting constitutive internalization mechanisms. Then, cytotoxicity was evaluated after incubation for 30 min of Dox-loaded Apt-Dend-NP-Dox, followed by wash and the determination of cell viability after 24 h. Not surprisingly, Apt-Dend-NP-Dox shows excellent selectivity: the viability of control cells (NIH-3T3) was less affected by the treatment and was greater than 60% compared to the control at all the tested concentrations (up to 20 µM of Dox). Conversely, cytotoxicity in MIA PaCa-2 cells is significantly higher, with a clear dose–response trend and residual 15% viability at 20 µM Dox concentration (Figure 3b).

**Figure 4:**
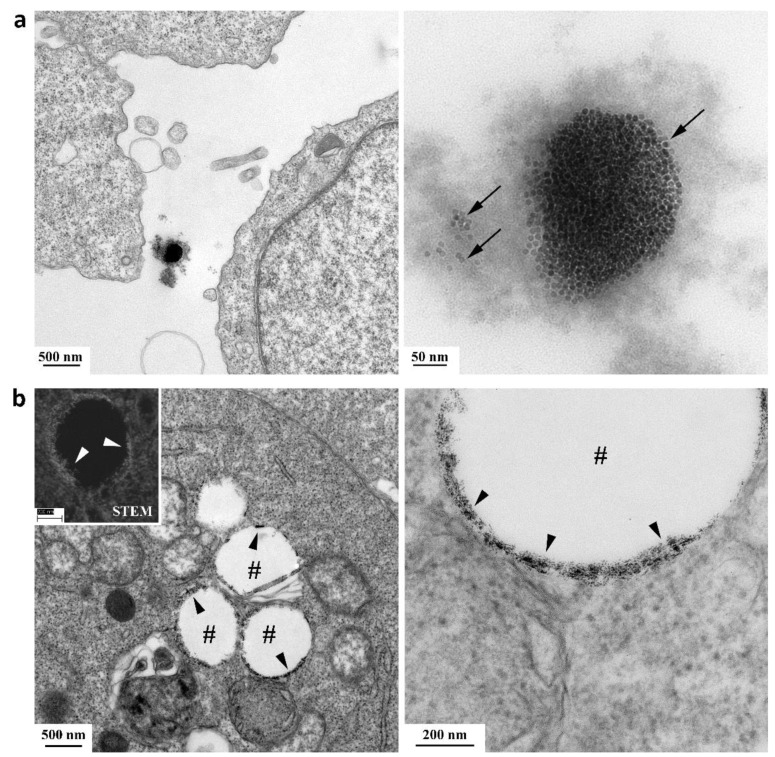
Ultrastructural analysis of Apt-Dend-NP inside MIA-PaCa-2 cells. (**a**) Metal aggregates found in the extracellular medium; (**b**) Dendrimer internalization by micropinocytosis mechanism. Arrows = SPIONs aggregate. Arrowheads = Apt-Dend. # = macropinosomes.

We finally sought to better investigate the endocytosis process of our PAMAM-coated magnetic nanoparticle with transmission electron microscopy (TEM) (Figure 4). While PAMAM-coated SPIONs are reportedly readily internalized without losing their organic dendrimer coating [8], we observed that our modified architecture shows a peculiar cell compartmentalization. Indeed, the magnetic core of the nanoparticle is found in larger aggregates outside the cell (arrows in Figure 4a) after 1 h of treatment, while dendrimer and drug payload are found in large internalized vesicles [28], presumably macropinosomes (arrowheads and number in Figure 4b) [29,30,31]. Based on the proven stability of dendrimer–nanoparticle assembly in cell culture medium [7], the observed cell selective cytotoxic activity (Figure 3), and the possibility of performing magnetically enhanced nanoparticle internalization (Figure 2), we can confidently rule out the hypothesis of nanoparticle disassembly in the medium. Conversely, it is likely that disassembly at the cell surface occurs, in which the delicate equilibrium between the lipid-coated dendrimer and magnetic nanoparticle is altered upon interaction of the targeting peptide with TfR receptor, leading to selective internalization of the organic portion with concomitant collapse of the metallic nanoparticle. Note that macropinocytosis is reported, together with clathrin-mediated endocytosis, as a preferential internalization pathway for dendrimers [32]. This result highlights the need for nanotechnologists to carefully evaluate with orthogonal techniques the internalization processes of all components when dealing with self-assembled structures. In fact, simply evaluating the serum stability and biologic activity of the payload could lead to a misinterpretation of nanoparticle behavior. Conversely, the observed disassembly at the cell surface could be exploited in the development of new multistage magnetic vectors that selectively deliver the payload without leading to undesired uptake of unnecessary—and potentially toxic—components.

## 3. Materials and Methods

### 3.1. Peptide Synthesis

Peptide T-7: HAIYPRH-amide was prepared by solid-phase synthesis using fluorenylmethoxycarbonyl (Fmoc) chemistry on an automatic Liberty Blue Peptide Synthesizer with an integrated microwave system (CEM, NC, USA). High-performance liquid chromatography (HPLC) analysis and purification were performed on a Dionex Ultimate 3000 PLC system with an autosampler. The crude peptide was purified by Reverse-Phase HPLC on a Jupiter 4 µm Proteo 90 Å column (250 × 10 mm; Phenomenex) using these solvents: water:TFA 100:0.01 *v/v* (eluent A)/acetonitrile:water:TFA 95:5:0.01 *v/v* (eluent B), flux 5 mL/min. The identity of the purified product was confirmed by electrospray mass spectroscopy, using an API3200QTRAP Hybrid Triple Quadrupole/Linear Ion Trap (ABSciex, Foster City, CA, USA).

### 3.2. Derivatization of PAMAM Dendrimer

First, 50 mg of PAMAM dendrimer G4 (25% C12) labeled with rhodamine (labeling degree: 1.35 mol Rhod/dendrimer) were dissolved in anhydrous DMSO (100 µL) and 10 equivalents of carboxy-derivatized peptide were dissolved in 50 µL of DMSO were added to the solution. *N*,*N*′-Diisopropylcarbodiimide (DIC, 11 equivalents) and *N*,*N*-diisopropylethylamine (DIPEA) were added to the reaction mixture, and the solution was stirred at 30 °C overnight. Next, 200 equivalents on succinic anhydride were added to the reaction mixture, and the solution was stirred for an additional 12 h. Finally, the solution was diluted with 600 µL of water and dialyzed extensively against water. The product was freeze-dried and stored at −20 °C until use.

### 3.3. Synthesis and Loading of Hybrid Nanoparticles

Dendrimer and Apt-Dendrimer-coated nanoparticles were synthesized according to a recently published procedure and purified by repeated magnetic washings [7,8]. The loading of coumarin 6 or doxorubicin was performed by incubating for 12 h the nanoparticle solution in phosphate-buffered saline (PBS) solution in the presence of an excess of payload. Briefly, a 10 mg/mL solution of coumarin 6 or doxorubicin in the appropriate solvent (DMSO for coumarin 6, water for doxorubicin) was mixed in 1:1 ratio with a 2.6 nM aqueous solution of nanoparticles (NP concentration calculated on the basis of Fe equivalents, 30.1 µM). An identical volume of PBS was added, and the solution was stirred overnight. Next, loaded nanoparticles were separated from the excess payload by magnetic washings. Briefly, suspension of nanoparticles was allowed to settle in the presence of a magnet placed under the container, and the supernatant was carefully removed by pipetting. The process was repeated until the supernatant was completely clear. In the case of coumarin 6 loading, nanoparticles were further purified by elution in PBS on a Sephadex G100 column (Sigma Aldrich, St.Louis, MO, USA).

### 3.4. Measurement of Doxorubicin Release in Cuvette

Dox-loaded nanoparticles were diluted in a buffered solution (citrate/phosphate 20/60 mM) at pH 7.4. The system was equilibrated at 25 °C and fluorescence emission was measured with λ_exc_ = 480 nm and λ_em_ = 500–600 nm. Next, pH of the solution was changed with sequential additions of HCl 1M, and pH was measured after each addition using a pH-meter (pH electrode, PH 6 XS, XS Instruments, Modena, Italy). Fluorescence emission was measured after equilibrating the solution for a minimum of 15 min.

### 3.5. DLS Measurements

Measurements were performed in standard capillary cells DTS 1060 (for z-potential measurements) or a 50 µL standard cuvette (for size measurements) on a Malvern Zetasizer nano ZS90 (Malvern Panalytical, MAlvern, UK), following the manufacturer instructions.

### 3.6. Cell Culture

Human pancreatic carcinoma cells (MIA PaCA-2) and mouse embryonic fibroblast cells (NIH-3T3) were purchased from the American Type Culture Collection (ATCC). Both cell lines were grown using a previously reported protocol. Briefly, both cell lines were maintained in Dulbecco’s Modified Eagle’s Medium (DMEM) with high glucose concentration (4.5 g/L) and supplemented with 10% fetal bovine serum (FBS), 4 mM L-glutamine, 1 mM sodium pyruvate, 100 U/mL penicillin, and 100 mg/mL streptomycin (Invitrogen). Cells were maintained at 37 °C in a humidified 5% CO_2_ atmosphere.

### 3.7. Confocal Imaging of Cells

Cells were imaged using a Leica TCS SP5 SMD inverted confocal microscope (Leica Microsystems AG, Wetzlar, Germany) interfaced with a diode laser (Picoquant, Berlin, Germany) for excitation at 405 nm, with Ar lasers for excitation at 488 and 561 nm. Glass-bottom Petri dishes containing cells were mounted in a thermostated chamber at 37 °C (Leica Microsystems) and viewed with a 63 × 1.2 NA water immersion objective or 40× 1.5 NA oil immersion objective (Leica Microsystems). The pinhole aperture was set to 1.0 Airy. All data collected were analyzed by ImageJ software version 1.44o.

### 3.8. Assessment of Cellular Uptake by Confocal Microscopy

Internalization, cellular uptake, and the release of fluorescent payload assays were performed according to established protocols. MIA PaCa-2 cells were seeded 24 h before the experiment in WillCo dishes to reach 80%–90% confluence. Standard conditions for incubation consisted of 30 min of incubation at 37 °C, 5% CO_2_ in DMEM. After incubation, cells were washed three times with PBS, fresh serum-containing medium was added, and the sample was imaged by confocal microscopy.

### 3.9. WST-8 Cell Viability Assay

The cytotoxicity of Dox-Apt-NPs complexes was evaluated by using a tetrazolium salt, 2-(2-methoxy-4-nitrophenyl)-3-(4-nitrophenyl)-5-(2,4-disulfophenyl)-2*H* tetrazolium, monosodium salt (WST-8) assay. MIA PaCA-2 cells or NIH-3T3 cells (1 × 10^4^ cells per well) were seeded in 96-well plates. After culturing for 24 h, the cells were incubated with a 2% serum-containing medium containing nanoparticles at a Dox concentration ranging from 0 to 30 µM for 1 h. After the incubation, the medium was removed, and cells were incubated with WST-8 reagent (10 µL) and 2% serum-containing medium (90 µL) for 2 h. Absorbance (450 nm) was measured using a microplate reader (Glomax Discovery, Promega, Madison, WI, USA) following producer instructions. The percentage of cell viability was determined by comparing drug-treated cells with the untreated cells (100% viability). Data represent the average of three independent experiments. Error bars represent the SD from three independent experiments.

### 3.10. MIA PaCa-2/Targeted Nanoparticles Interaction Ultrastructural Analysis

Targeted nanoparticles internalization in MIA PaCa-2 cells was evaluated with TEM analysis. Not treated and 1 h-treated cells were fixed using 1.5% glutaraldehyde solution, followed by 2% osmium tetroxide. Then, pellets were stained with 3% uranyl acetate, dehydrated, and embedded in epoxy resin (Epon 812, EMS), which was cured for 48 h at 60 °C [33]. Samples were sectioned in 90-nm thin slices using an ultramicrotome (UC7, Leica Microsystem, Wetzlar Germany) equipped with a 45° diamond knife (DiATOME, Nidau, Switzerland), and sections were collected on 300-mesh copper grids (EMS). The ultrastructural analysis was performed using a Zeiss Libra 120 Plus microscope, operating at 120 kV and equipped with an in-column omega filter, in bright field and scanning transmission electron microscopy (STEM) mode.

## 4. Conclusions

In conclusion, we assembled a new metal–organic hybrid system potentially suitable for theranostics applications. Our assembly involves a magnetic core suitable for in vivo imaging, a pH-responsive PAMAM dendrimer able to adsorb—and release in mildly acidic conditions—a therapeutic molecule, and a surface functionalization that inhibits nonspecific internalization processes while promoting targeted internalization in MIA PaCa-2 cells. Additionally, the presence of a SPION core allows the magnetically enhanced internalization in cells. A further peculiar property of this system is represented by its ability to disassemble at the surface of the cell, leading to the selective internalization of the organic portion of the nanostructure. Far from being a limitation, this peculiar behavior suggests an innovative use of hybrid organic–metallic drug delivery systems. Indeed, it would be theoretically possible to perform a magnetically driven accumulation of drug-loaded nanoparticles, followed by selective internalization of the drug–organic part. The metallic core—which remains on the outer side of the cell—is sufficiently small to be cleared by the organism without leading to unwanted accumulation effects [34]. Further studies are in progress to implement the behavior of structurally similar systems for theranostic and magnetically driven therapy purposes.

## Supplementary Materials

The following are available online at https://www.mdpi.com/1420-3049/25/9/2252/s1, Figure S1: Targeted nanoparticles internalization in cells.
