# Supplementary material for: Targeted Dendrimer-Coated Magnetic Nanoparticles for Selective Delivery of Therapeutics in Living Cells
Source: Molecules. 2020 May 10;25(9):2252. doi: 10.3390/molecules25092252 (PMC7249066; doi:10.3390/molecules25092252)
Supplement: Supplementary file 1 [file molecules-25-02252-s001.pdf]

SUPPLEMENTARY INFORMATION

Targeted dendrimer-coated magnetic nanoparticles for selective delivery of therapeutics in living cells

Paola Parlanti, Adriano Boni, Giovanni Signore Melissa Santi

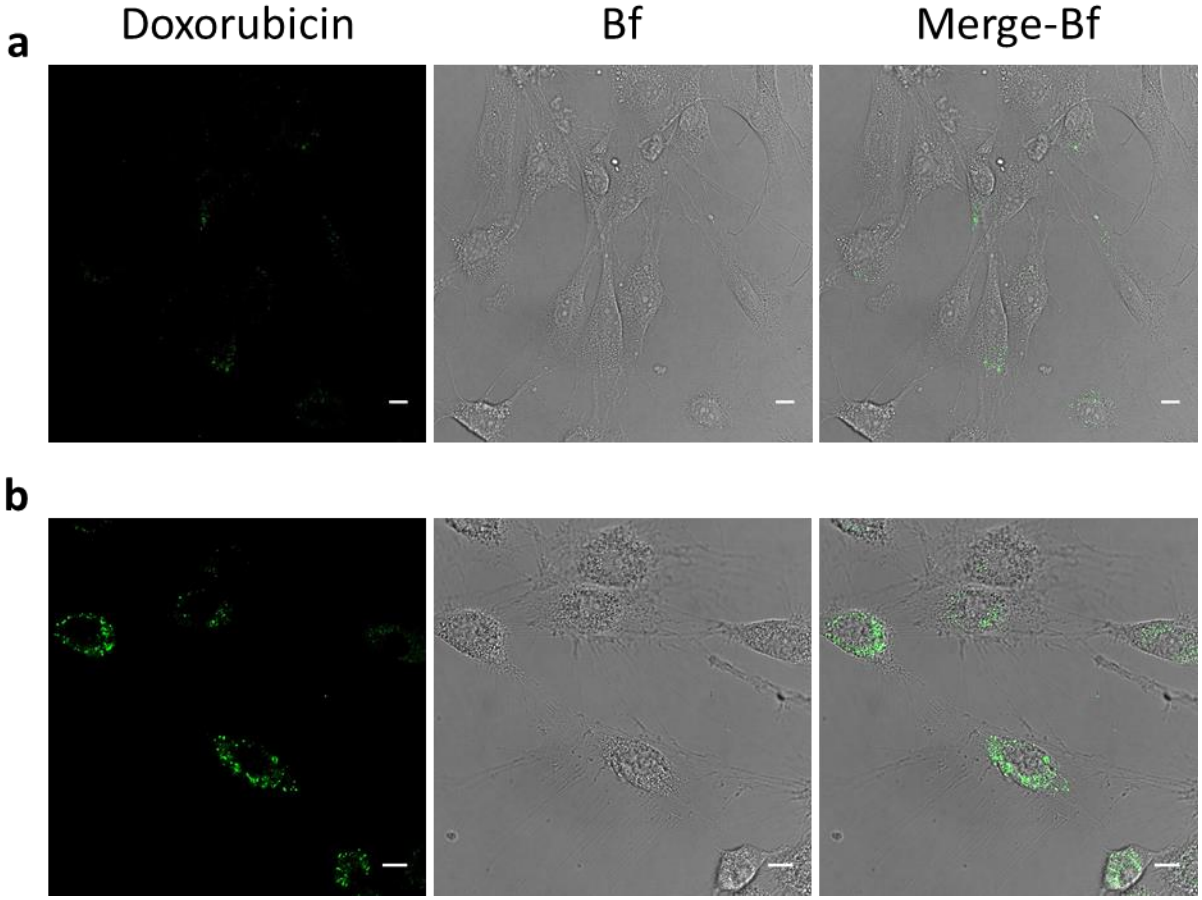

Figure S1: Targeted nanoparticles internalization in cells. MIA-PaCa-2 cells were treated with Dend-NP-Dox untargeted (a) and Apt-Dend-NP-Dox targeted (b) nanoparticles for 2 h at 37 °C and then imaged by confocal microscopy. Scale bar: 10 µm.
